# Synthesis and Photochemical Properties of Monolithic TiO_2_ Nanowires Diode

**DOI:** 10.3390/molecules26123636

**Published:** 2021-06-15

**Authors:** Massimo Zimbone, Maria Cantarella, Giuliana Impellizzeri, Sergio Battiato, Lucia Calcagno

**Affiliations:** 1 CNR-IMM, Via Santa Sofia 64, 95123 Catania, Italy; massimo.zimbone@ct.infn.it (M.Z.); maria.cantarella@ct.infn.it (M.C.); giuliana.impellizzeri@ct.infn.it (G.I.); 2 Department of Physics and Astronomy, University of Catania, 95123 Catania, Italy; lucia.calcagno@ct.infn.it

**Keywords:** nanowires, TiO_2_, photocatalysis, photovoltage, water treatment

## Abstract

In this paper, the structural and photochemical properties of a monolithic photochemical diode are discussed. The present structure is composed, from the top to the bottom, of a TiO_2_ nanowire layer, a TiO_2_ film, a Ti foil, and a porous layer made of Pt nanoparticles. The synthesis of the nanowires was simply carried out by Au-catalysed-assisted process; the effects of the annealing temperature and time were deeply investigated. Morphological and structural characterizations were performed by scanning electron microscopy and Raman spectroscopy. The analyses showed the rutile structure of the TiO_2_ nanowires. The photocatalytic properties were studied through the degradation of methylene blue (MB) dye under UV light irradiation. The nanowires induced an enhancement of the photo-degradation rate, compared to TiO_2_ in a bulk form, due to an increase in the surface area. Moreover, the presence of a nano-porous Pt layer deposited on the rear side of the samples provided a further increase in the MB degradation rate, related to the scavenging effect of Pt nanoparticles. The overall increment of the photo-activity, due to the nano-structuration of the TiO_2_ and to the presence of the Pt layer, resulted a factor 7, compared to the bulk reference. In addition, photovoltage measurements allowed to assess the effects of TiO_2_ nano-structuration and Pt nanoparticles on the electron accumulation.

## 1. Introduction

One of the major concerns of the humanity is the supply of water. The World Health Organization estimated that, by 2025, half of the world’s population will reside in areas with high water stress [1]. The scarcity of clean water could be mitigated by a better management of the water resources, but also by the development of novel materials and methodologies able to efficiently purify wastewater. Conventionally, the technologies used for water treatment are sedimentation, adsorption, filtration, or inverse osmosis. These methods are constrained by the high operating costs and high energy consumption, which pose serious questions regarding their use in poor countries. Consequently, the finding of new materials able to recycle wastewater efficiently, and in an eco-friendly manner, is highly urgent [2].

This problem has recently aroused a considerable interest from the scientific community. In this context, the heterogeneous photocatalysis could solve the above-considered problem efficiently and with low cost. The rationale of the photocatalytic process is based on the production of reactive oxygen species (ROSs), such as OH• and O_2_•−, active in the mineralization of organic contaminants and elimination of water pathogens [1,3,4]. In a photocatalytic semiconductor, such as TiO_2_, the production of ROS is driven by the absorption of photons and the following formation of electron-hole couples. The ROSs promote oxidation processes, which destroy organic compounds and damage microorganisms possibly present in water [5,6,7]. Photocatalysis can be regarded as a definitive, and eco-friendly, method for the degradation of contaminants and reduction in bacteria load from wastewater.

The main technological drawbacks hindering the commercialization of the photocatalysts are the low efficiency of the process and the need for photocatalytic powders recovery after the wastewater treatment. The former is due to intrinsic factors and can be overtaken by properly controlling the quality of the material [8], while the latter can be solved by anchoring the photocatalyst to a substrate. As concerns the efficiency of the material, the most prominent issues to be solved are:

1. the presence of recombination channels that reduces the electron-hole flux through the liquid-solid interface;
2. the small liquid-solid interface area;
3. the low photon absorption in the visible range, caused by the high bandgap of TiO_2_ (~3.2 eV), that restricts its use in only a narrow region of the solar spectrum (about 7%).

Several strategies can be adopted to overcome these limitations and to improve the photocatalytic performances. For example, the use of noble metals (e.g., Pt or Au) enhances the photoactivity by improving the charge separation of electrons and holes, through the formation of a Schottky junction at the metal/semiconductor interface [9,10,11,12,13]. Nano-structuration of the photocatalyst, and, in particular, the formation of nanowires, can be considered an effective pathway to increase the water-TiO_2_ contact area and to reduce the time needed for the charges to reach the surface (preventing detrimental electron-hole recombination) [14,15,16,17,18].

In the present article, we used the above-mentioned strategies to improve the photocatalytic efficiency of TiO_2_ photocatalyst. In detail, we synthesized TiO_2_ nanowires (NWs) of several μm in length with large surface area and high absorption aptitude in the UV-visible range. The nanowires were capped with Au nanoparticles (NPs), while Pt NPs were deposited on the rear side of the samples, as to take the advantages of the electron-scavenging effect of the noble metals. The realized photochemical diode showed promising abilities in the application field of water treatment.

## 2. Materials and Methods

Titanium foils, with a thickness of 0.25 mm and purity of 99.99% (Sigma Aldrich, Darmstadt, Germany), were used as substrates for the growth of the TiO_2_ nanowires. The Ti foils were cut into pieces of 1 cm × 1 cm, and then a thin gold layer was deposited on the sample surface using an RF (60 Hz) Emitech K550X sputter. The Au target had a 99.999% purity, and the deposition was carried out in an Ar flow at a current of 10 mA and a chamber pressure of 0.02 mbar. The Au thickness, evaluated by Rutherford Backscattering Spectroscopy (RBS) using a 2.0 MeV He^+^ beam and a 165° scattering angle, was 5 nm. The samples were then inserted in a covered quartz holder and TiO_2_ NWs synthesis was performed in air in a conventional furnace. Samples were heated at a heating rate of 4 °C/min until the final temperature was reached. The annealings were performed in the temperature ranges of 700–900 °C and annealing time in the range of 2–4 h. Samples without Au layer were also grown as reference.

The synthesis of platinum nanoparticles (Pt NPs) was performed by pulsed laser ablation (PLA) in liquid with the same experimental apparatus described in Ref. [11]. Platinum metal plate (purity 99%) was purchased by Sigma Aldrich, Darmstadt, Germany. Nanoparticles, dispersed in water, resulted stable for some months. Pt NPs were 20 nm in diameter (as measured by dynamic light scattering) and have a surface/mass ratio of about 20 m^2^/g [12].

Monolithic chemical diodes were manufactured as reported in Ref. [12]. Briefly, the diodes were realized by sanding (with a sandpaper) the backside of the titanium foils (roughing the back-surface), then depositing several ml (drop by drop) of the Pt NPs solution at 90 °C and waiting until the complete water evaporation. Thus, a continuous porous layer of Pt NPs on the titanium was realized.

SEM analyses were conducted with a Gemini field emission SUPRA 25 Carl Zeiss microscope. Total reflectivity was carried out using a Lambda 40 Perking-Elmer spectrophotometer.

Raman spectroscopy was performed by a Horiba Jobin Yvon HR550 system equipped with a 633 nm HeNe laser, with a resolution of 0.2 cm^−1^. The power of the laser was kept below 1 mW, and an objective of 40× with 0.75 NA was used. For each sample, four different areas were analyzed, and three spectra were acquired for each area, using a collection time of 60 s.

The photocatalytic properties were studied by monitoring the degradation of the methylene blue (MB) dye, in de-ionized water solution under UV light irradiation. A TL 8W BLB Philips UV lamp, centered at 368 nm (wavelength in the range 350–400 nm) was used as light source. The irradiance of 1.1 mW/cm^2^ was measured at the sample surface. The samples were placed vertically in a closed cuvette with a MB solution at a concentration of 1.3 10^–5^ M, (which corresponds to a 1 optical density). The concentration of the dye was monitored “online” with a homemade equipment by measuring the solution transmittance in the wavelength range of 630–700 nm. The equipment allowed the measure of 6 different cuvettes at the same time. Before the photocatalytic test, the samples were kept in dark for 2 h for allowing the dye to adsorb on the surface of the samples and cuvettes; then the UV light was switched on for a total time of 6 h. Under UV light irradiation, the MB concentration usually decreases according to a first-order kinetic law: C = C_0_exp(–Γt), where *C* is the time dependent concentration of MB, C_0_ is the initial MB concentration, *Γ* is the discoloration rate constant, and *t* is the time expressed in hours [2].

Photovoltage measurements were performed using a Versastat-4 potentiostat in a three electrodes setup with Pt wire as the counter electrode, a saturated calomel electrode (SCE) as the reference electrode, and the samples as the working electrode. The measurements were performed at room temperature and atmospheric pressure, in a one-compartment electrochemical cell filled with 100 mL of ultrapure water.

## 3. Results and Discussion

**Figure 1:**
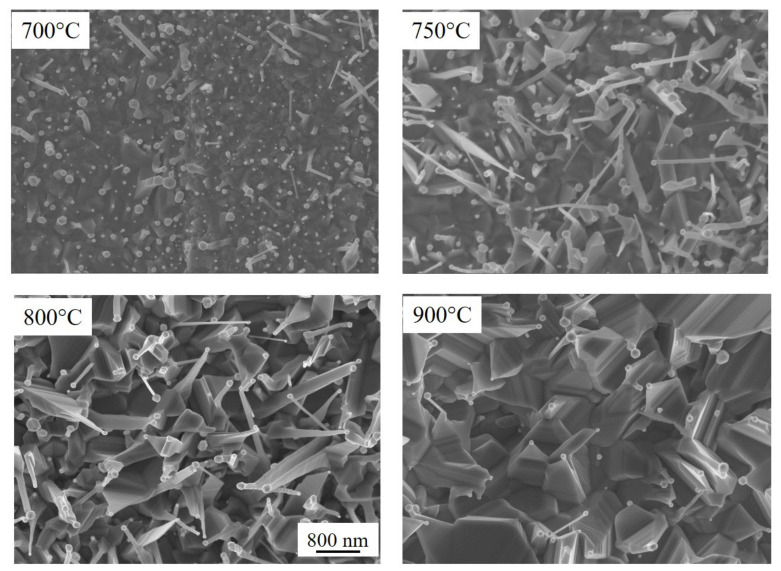
SEM images in plan-view of the TiO_2_ NWs grown at different temperatures for a fixed annealing time of 4 h. Marker is the same for all images.

In order to investigate the morphology of the TiO_2_ NWs, the Ti foils, coated with a thin film of Au, were annealed in air at several temperatures (700–900 °C) and annealing times (2–4 h). Figure 1 shows the SEM images of the sample surface after four hours of thermal treatment at different temperatures: from 700 up to 900 °C.

The sample annealed at 700 °C displays the gold cluster formation on the surface, while few TiO_2_ nanowires are produced. The formation of gold clusters, induced by the thermal annealing, can be ascribed to the well-known gold de-wetting process [19]. Upon increasing the temperature to 750 °C, the presence of cylindrical NWs can be observed. It is worth noting that the NWs have a gold nanoparticle on top; this feature can be related to the NWs formation process [20]. Indeed, Ti diffusion from the substrate to the interface between TiO_2_ and Au is the driving force for the formation of the nanowires, being the Au able to catalyze the NWs growth [21,22]. At 800 °C, TiO_2_ nanorods are formed and show faceting: only low energy facets appear due to the crystalline structure of the NWs. A further increase in the temperature determined the formation of more faceted NWs. Indeed, at 900 °C the faceting is pronounced and the structures are larger. Moreover, only the rectangular-shaped NWs can be now observed, while the cylindrical ones are no longer present. Figure S1 shows a high magnification of the nanowires. Here, it is evident that, at 750 °C, the NWs are cylindrical while at 800 °C they have more faceted structures. Figure S2 shows a low magnification of the samples. A high density of nanowires is clearly observed for samples annealed at 750 °C and 800 °C.

**Figure 2:**
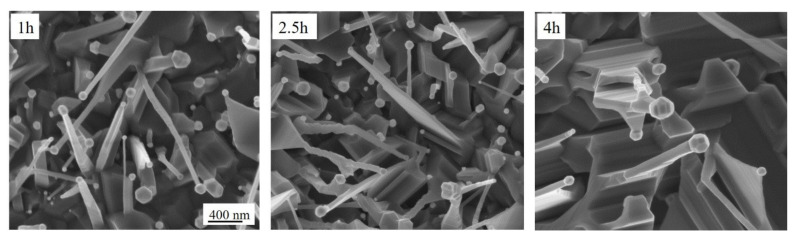
SEM images in plan-view of the TiO_2_ NWs grown at 800 °C for different annealing times.

Figure 2 displays the SEM images of the samples annealed at 800 °C for different times: from 1 to 4 h. After 1 h annealing, the TiO_2_ NWs have a cylindrical shape, while for higher annealing time, the shape changes from a cylindrical one to facet and trapezoidal one. Another interesting feature is the faceting of the Au nanoparticle after 4 h of thermal treatment.

Figure S3 shows a low magnification of the samples annealed at 800 °C for different times.

**Figure 3:**
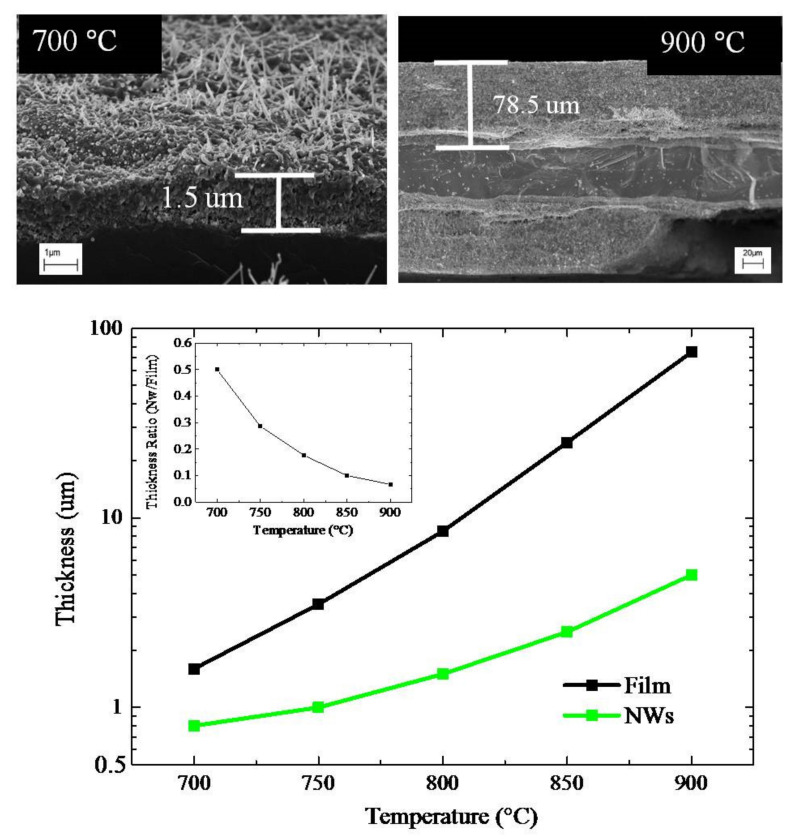
SEM images in cross-view of the samples annealed at 700 and 900 °C (**top**). The thickness of the underneath layer and the length of the nanowires as a function of annealing temperatures for an annealing time of 4 h (**bottom**). The inset reports the ratio between nanowires length and interlayer thickness as a function of the annealing temperature.

Figure 3 (top) presents the cross-section SEM images of two samples annealed at 700 or 900 °C for 4 h. The annealing in air of the thin Au film on Ti foils produced the TiO_2_ NWs, as well as an oxide interlayer between the NWs and the Ti substrate. In particular, an oxide film, with a thickness of about 1.5 μm and 78.5 μm is observed for the samples annealed at 700 and 900 °C, respectively. The interlayers were porous and polycrystalline, and were composed of grains with different sizes. In detail, the samples annealed at 750°C for 4 h showed grains of 100 nm in size (see Figure S4). The thicknesses of the interlayer and the length of the NWs were estimated and reported in Figure 3 (bottom). Note the logarithm ordinate scale. In the inset, the ratio between the NWs length and the interlayer thickness was reported in a linear scale.

**Figure 4:**
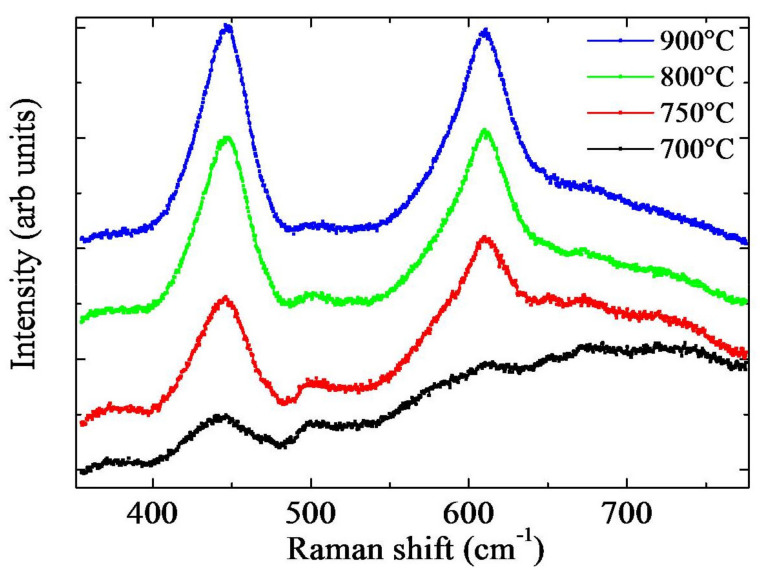
Raman spectra of TiO_2_ NWs annealed at different temperatures for 4 h.

Raman measurements were also performed to investigate the crystalline structure and quality of the TiO_2_ NWs. In Figure 4, the spectra of TiO_2_ NWs annealed at different temperatures for 4 h are reported. The rutile phase is apparent for all the produced samples, as inferred by the presence of the two peculiar peaks at 445 and 610 cm^-1^. These peaks are classified, in literature, as E_g_ and A_1g,_ respectively [23]. The peaks’ intensities further increase as the temperature approaches 900 °C. This monotonic increase can be due to longer nanowires and higher film thickness as observed in Figure 3 but also to a larger crystallite size. By comparing the NWs and the thermal oxide film spectra, another small peak at 500 cm^−1^ and a broad structure at 700 cm^−1^ appeared for NWs, as reported in Figure S5. These small features could be due to relaxation of Raman selection rules, owing to the small dimension of the nanowires or to the presence of surface optical modes. Nano-structuration of the material usually also influences the absorbance of the samples. In Figure S6 two absorption spectra of a sample with the TiO_2_ NWs and a reference film, both obtained at 750 °C for 4 h, are reported. The increase in the absorbance at 400 nm is related to the TiO_2_ bandgap absorption, while higher wavelength absorption should be related to the Ti substrate. The absence of interference fringes means that even the reference film without nanowires is rough. Nano-structuration of the surface leads to an increase in the absorbance from 82% to 91% at 368 nm.

Summing up, the samples annealed at 750 °C for 4 h, produced a good crystalline quality, good NWs coverage, and high value of the ratio between NWs length and interlayer thickness. In particular, the length of the TiO_2_ NWs was about 1 μm, while the thickness of the interlayer was 4 μm, giving back a ratio of about 0.25. At 700 °C, the NWs showed a low density (see Figure 1), an undefined phase (see Figure 4), and a small oxide thickness (see Figure 3). At 800 °C, the NWs were faceted and showed a high density. Despite they undoubtedly possessed good crystalline quality, as revealed by the Raman measurements (Figure 4), a thick oxide layer was formed (Figure 3). This can be detrimental, indeed, as a thin oxide would minimize the resistivity of the samples, promoting the electron transfer to the rear side of the sample. On the basis of these results, the samples annealed at 750 °C for 4 h were chosen for the photocatalytic tests.

**Figure 5:**
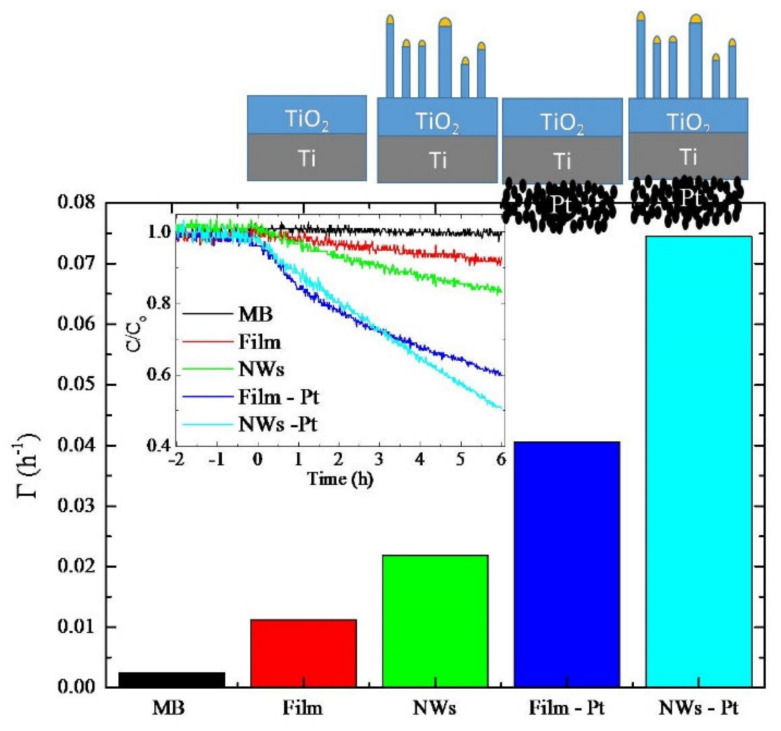
Photocatalytic activity of different samples: MB (without any catalyst), film, TiO_2_ NWs, film with Pt, and NWs with Pt.

The photoactivity of the samples was evaluated by MB dye degradation. In the inset of Figure 5, we reported the degradation of MB as a function of the irradiation time. Negative time values refer to the preconditioning test in which the MB solutions with the samples were tested in the dark. During this period time, the MB concentration did not show a significant decrease, indicating a negligible absorption of MB onto the sample holder and the sample surface. Then, the UV light was switched on. The slopes of the curves reported in the inset represent the pseudo-first-order photocatalytic rate constants. The discoloration rates (i.e., the slope values) were reported in Figure 5 for the different samples: “MB” refers to MB solution without any catalyst; “Film” indicates the reference sample obtained by annealing a Ti foil at 750 °C for 4 h; “NWs” refers to the sample annealed in the presence of gold nanofilm; “Film-Pt” indicates the reference sample with the Pt nanoparticles deposited on the back of the specimen; “NWs-Pt” refers to the sample with the TiO_2_ NWs and also the Pt NPs on the backside. Reference sample (“Film”) activity was estimated to be 1.1 × 10^-2^ h^−1^; the nano-structuration (“NWs”) increased the activity of about 2 times (2.2 × 10^-2^ h^−1^). The presence of the nano-porous Pt film (“Film-Pt”) on the rear side of the samples enhanced the photo-activity even more. Indeed, by the addition of Pt nanoparticles, the rate constant increased by a factor 3.5 in both samples, as evidenced by the measured activities of 4.0 × 10^−2^ h^−1^ and 7.4 × 10^−2^ h^−1^ for Film-Pt and NWs-Pt samples, respectively. Interestingly, the overall increase in the photo-activity, due to the nano-structuration and the presence of Pt, is a 6.7-fold factor.

By comparing the photocatalytic activity of the “Film” and the “NWs”, it is clear that the nano-structuration had a beneficial effect, which can be reasonably attributed to the higher electron transfer to the molecules in solution, and it can be due to several concomitant reasons:

1 the increase in the surface area allowing a higher photo-carrier exchange with the solution;
2 the improvement of the light absorption at 365 nm of about 10% (as observed in Figure S6);
3 the presence of gold nanoparticles on the top of the nanowires (Figure 1), that enhances the electron transfer to the solution, since the well-known property of Au to be a good electron scavenger [24,25].

These result in an enhancement of the MB degradation, as experimentally observed. An increase in the photoactivity is also obtained in the presence of the Pt nanoparticles on the rear side of the sample (the reader can compare the photo-activity of the “Film” and “Film-Pt” samples). This is ascribable to the scavenging effect of Pt nanoparticles; indeed, the photo-excited electrons were driven toward the electrode’s back contact. It has been largely demonstrated that the higher the electron scavenging effect is, the higher the photoactivity is [9]. Both the nano-structuration of the front surface, due to the TiO_2_ nanowires, and the presence of nano-porous Pt film on the back surface, positively influenced the photoactivity of the investigated materials.

**Figure 6:**
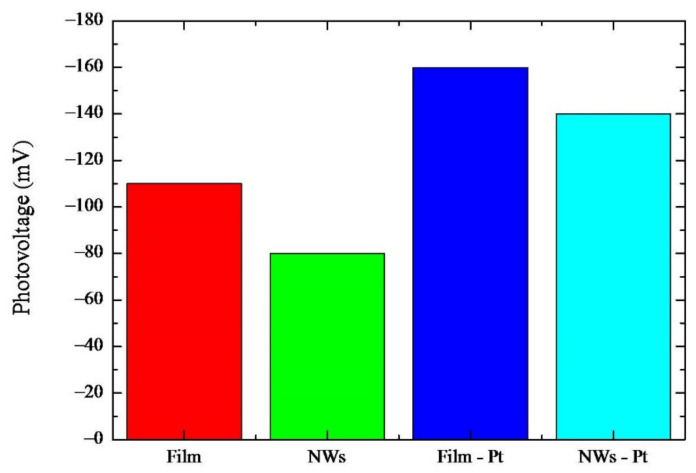
Photovoltage for various samples: “Film”, “NWs”, “Films-Pt”, and “NWs –Pt”.

In order to attain a better insight into the mechanisms of the photocatalysis in this peculiar system, we measured the photovoltage. Photovoltage is defined as the difference between the open-circuit voltage (VOC) in the dark and under UV light irradiation [26]. In Figure 6, the measurements for the four investigated typologies of samples are summarized. For all the samples, negative photovoltage values are found, pointing to an electron accumulation under illumination while holes, produced by the UV excitation in TiO_2_, were transferred to the solution [27].

An increase in VOC (in module) is observed once the irradiation is started, and it reaches a steady-state value after about 10 min of continuous illumination (not shown). The photovoltage measured in the “Film” sample was about −110 mV. Upon nano-structuration (see “NWs” sample), the photovoltage was reduced to −80 mV. Thanks to the presence of the nanoporous Pt film on the rear side of the samples an increase in the photovoltage was observed; indeed, “Film-Pt” and “NWs-Pt“ samples had photovoltage values of −160 mV and −140 mV, respectively.

The slight decrease in the photovoltage for the “NWs” with respect to the “Film” sample can be reasonably attributed to a decrease in the electron accumulation into the NWs samples. This effect is likely due to a higher electron dispersion into the solution in the case of the TiO_2_ NWs. It could be partly due to the electron scavenging effect of the gold particles on the top of the NWs, which counterbalances that of the Pt on the back side and/or allows a charge recombination effect. Concerning the increase in the photovoltage for the “Film-Pt” with respect to the “Film” sample, this indicates a higher electron accumulation due to the different structures of these samples. In particular, the “Film-Pt” sample was better able to collect electrons than the sample without Pt. Indeed, as previously reported, the Pt loading on Ti enriches the surface with O_2_ adsorption sites with adsorption energies up to −1.69 eV [10]. This facilitates the process of electron accumulation since the adsorbed oxygen species act as the primary electron acceptors, subsequently reacting with H^+^ to form HO_2_^−^ and/or H_2_O_2_ species [28]. Regarding the “NWs-Pt” sample in this case, an increase in the photovoltage, with respect to the sample, without Pt (“NWs”) was found. This sample (i.e., “NWs-Pt”) is very useful for evaluating the contribution of the two factors influencing the photovoltage, i.e., the nano-structuration and the Pt nanoparticles. Indeed, on one side, the nano-structuration is expected to increase the photovoltage, as described previously, whilst on the other, the Pt presence enhances the photovoltage. Finally, weighing these concomitant effects, the one given by the Pt layer proves to be predominant since the photovoltage for the “NWs-Pt” sample is slightly lower than the “Film” sample, where neither nano-structuration, nor Pt nanoparticles, are present.

Lastly, another thermodynamic consideration can be done by considering the value of the VOC reached by our samples under UV light irradiation. The VOC provides a valuable estimation of the position of the quasi-Fermi level of the majority carriers which indicates whether the photogenerated carriers are thermodynamically capable of a particular photo-electrolysis reaction. In our case, we observed a VOC of about −80 mV_SHE_ for all the samples. This means that some reactions, such as hydrogen evolution (H^+^/H_2_ E_0_ = −410 mV_SHE_, pH = 7) or oxygen reduction to superoxide (O_2_/O_2_^*-^ E_0_ = −286 mV_SHE_, pH = 7), are not thermodynamically allowed, while electrons can be consumed by other reactions, such as reduction in dissolved oxygen to hydrogen peroxide (O_2_/H_2_O_2_ E_0_ = 282 mV_SHE_ @pH = 7) or to water (O_2_/H_2_O E_0_ = 817 mV_SHE_, pH = 7) or to hydroxyl ion (O_2_/HO− E_0_ = −12 meV_SHE_ @pH = 7).

## 4. Conclusions

TiO_2_ NWs were simply obtained by thermal oxidation of Ti foils coated with a thin layer of Au. The thermal processes were performed at different temperatures and times, and the optimal growth parameters were fixed to 750 °C for 4 h. This process resulted in NWs with a good crystalline quality, good NWs coverage, and low value of the ratio between NWs length and interlayer thickness. Raman measurements of the nanowires revealed a TiO_2_ rutile crystalline phase formation. The analysis of the photocatalytic properties of the TiO_2_ nanowires reveals a higher degradation rate compared to a reference bulk. Moreover, the presence of the nano-porous Pt film on the backside of the sample provided a further increase in the degradation rate, related to the scavenging effect of the Pt nanoparticles. The overall increase in the photo-activity, due to the nano-structuration of the TiO_2_, and to the presence of the Pt layer, was about a factor of 7 with respect to the bulk sample, used as reference. In addition, the photovoltage measurements pointed to a decrease in the electron accumulation in the presence of the nano-structuration, and an increase induced by the Pt layer.

## Data Availability

the data present in this study are available on request from corresponding author.

## Supplementary Materials

The following are available online. Figure S1: High magnification SEM images in plan-view of TiO2 NWs grown at different temperatures for a fixed annealing time of 4 h, Figure S2: Low magnification SEM images in plan-view of TiO2 NWs grown at different temperatures for a fixed annealing time of 4h, Figure S3: SEM images in plan-view of TiO2 NWs grown at 800 °C for different annealing times, Figure S4: SEM image in cross-view of a TiO2 film grown at 750 °C for 4h, Figure S5: Comparison between the Raman spectra of NWs and the thermal oxide film grown at 800 °C for 4 h, Figure S6: Absorption spectra of “NWs“ and “Film” samples.

## Sample Availability

Samples of the compounds are available from the authors.
