# Supplementary figures and images for: Synthesis and Photochemical Properties of Monolithic TiO2 Nanowires Diode
Source: Molecules. 2021 Jun 15;26(12):3636. doi: 10.3390/molecules26123636 (PMC8232246; doi:10.3390/molecules26123636)

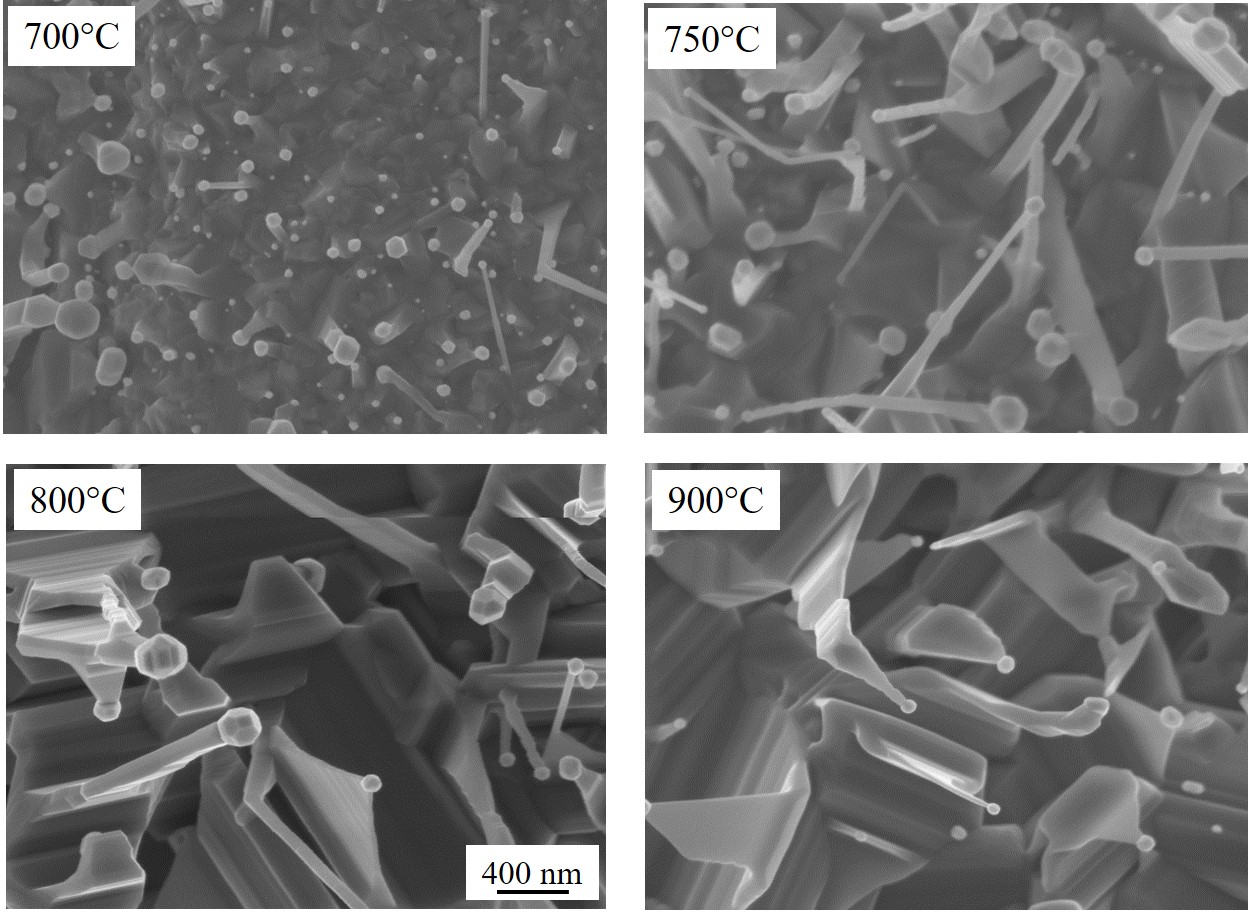

Supplement: Supplementary file 1 [file molecules-26-03636-s001.zip › Figure S1.jpg]

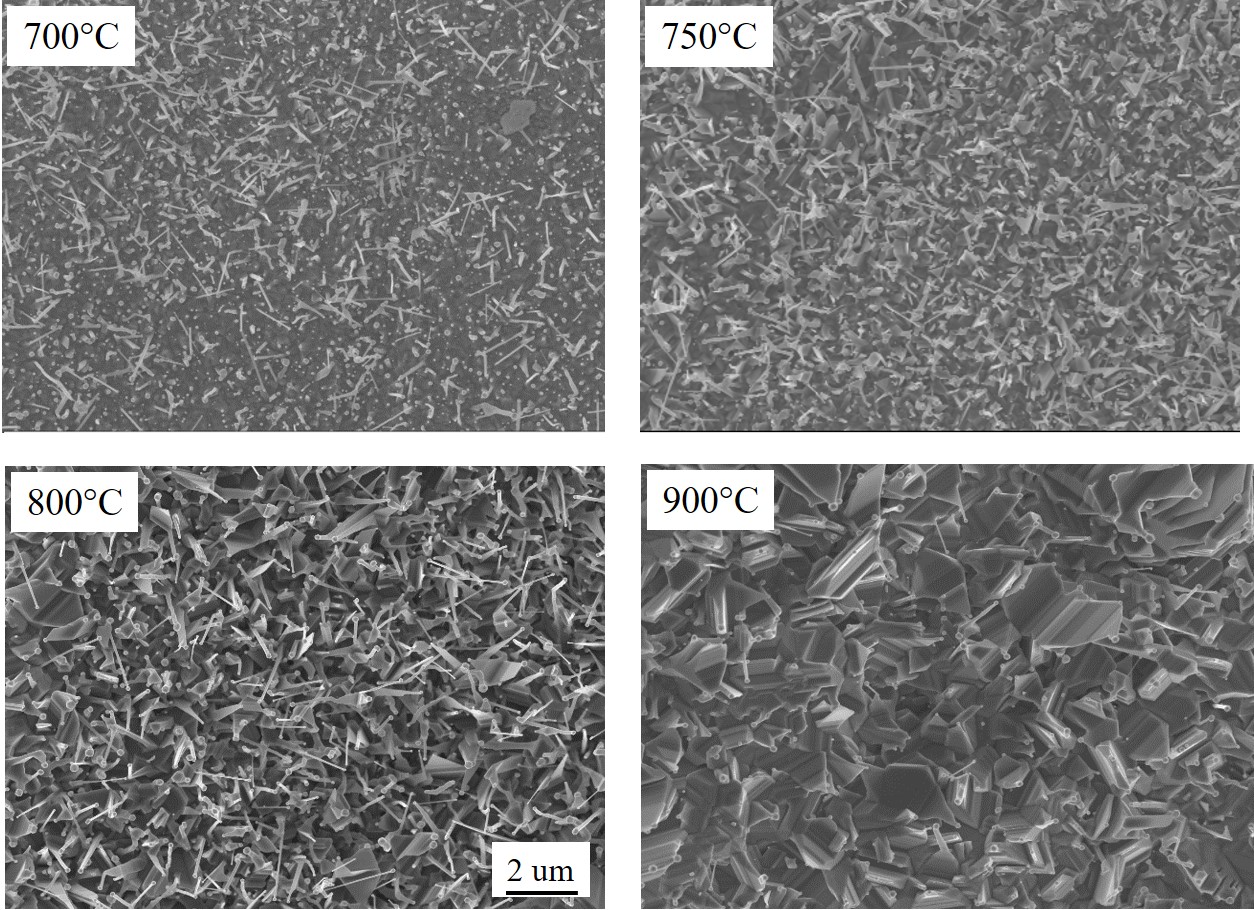

Supplement: Supplementary file 1 [file molecules-26-03636-s001.zip › Figure S2.jpg]

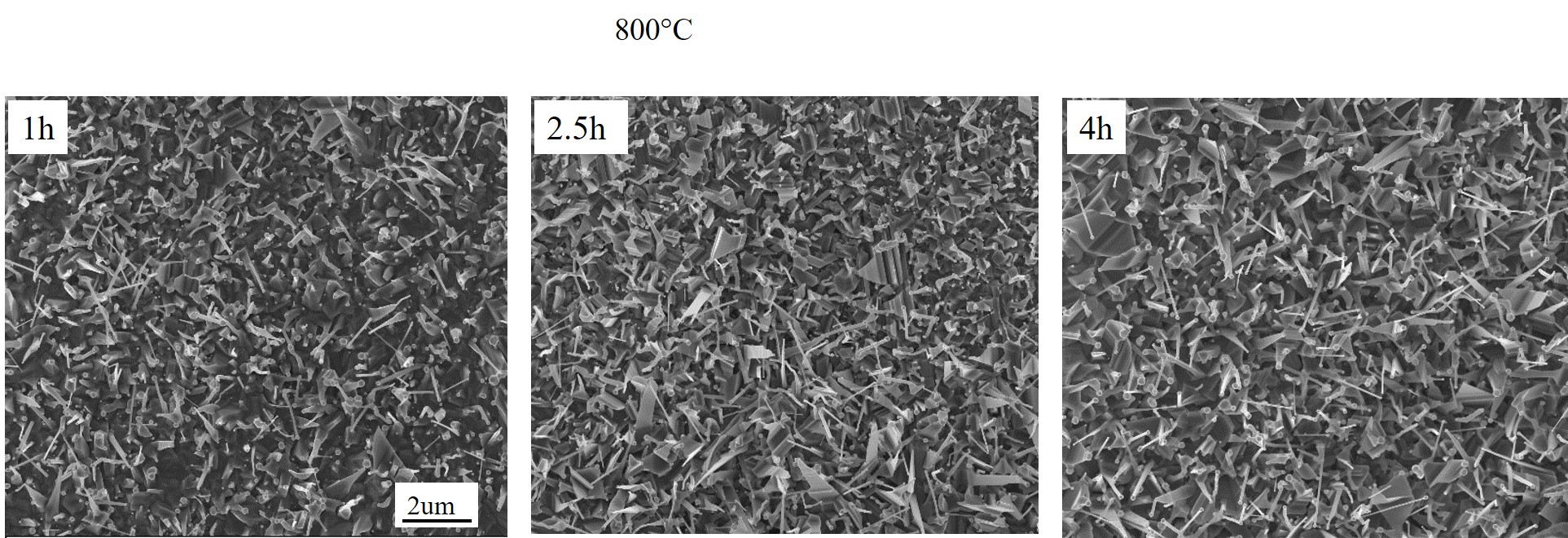

Supplement: Supplementary file 1 [file molecules-26-03636-s001.zip › Figure S3.jpg]

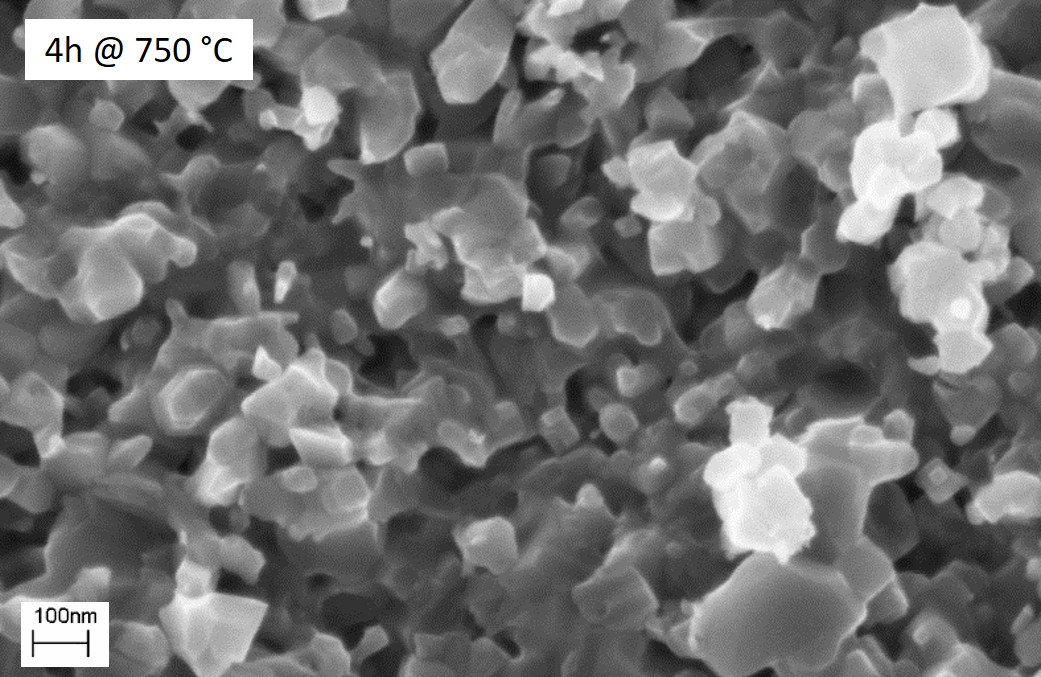

Supplement: Supplementary file 1 [file molecules-26-03636-s001.zip › Figure S4.jpg]

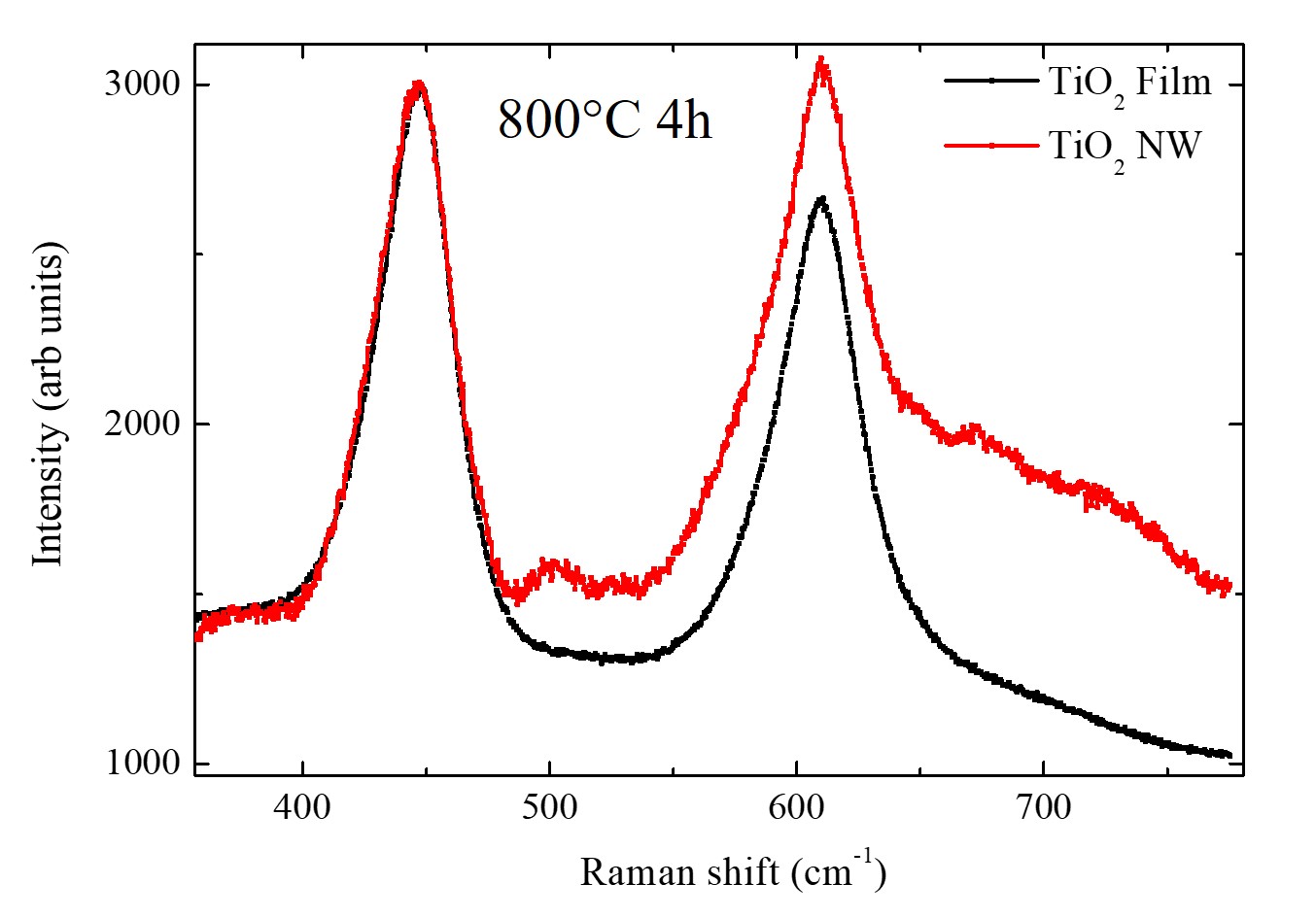

Supplement: Supplementary file 1 [file molecules-26-03636-s001.zip › Figure S5.jpg]

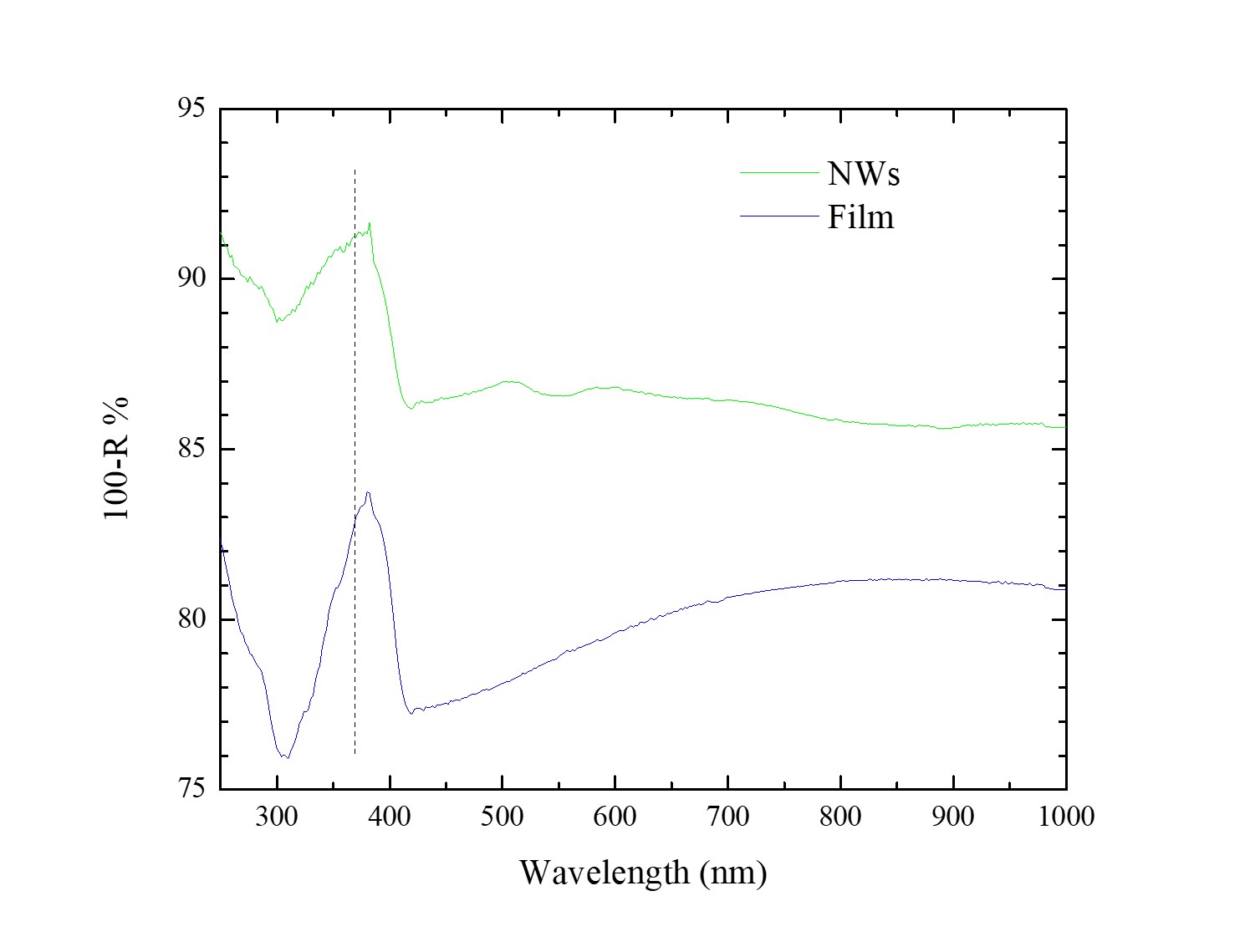

Supplement: Supplementary file 1 [file molecules-26-03636-s001.zip › Figure S6.jpg]
